# Observations on the Inoculated Cow-Pock

**Published:** 1801-07

**Authors:** T. M. Winterbottom

**Affiliations:** Physician of the Colony of Sierra Leone


### *Observations on the Inoculated Cow-Pock*,

By T. M. WINTERBOTTOM, M. D.
*Physician of the Colony of Sierra Leone.*

THE advantages which the Cow-pock possesses have of late very much excited the attention of the Public, more especially as its inoculation is safe, and practicable at every period of life, and almost in every state of health. In infancy, during the time of teething, when the irritability of the system is so much increased as to render the inoculated Small-pox dangerous and often fatal; likewise at an adult age, during the time of pregnancy, when the Small-pox is generally fatal to the infant, and often dangerous to the mother, the Cow-pock Inoculation may safely be employed. But when we reflect that the Cow-pock offers, at the same time, the means of annihilating that dreadful scourge the Small-pox, it evidently becomes our duty to spread the former as extensively as possible. There are, however, some circumstances respecting the inoculation for the Vaccine disease, which do not appear perfectly satisfactory. In a great number of the histories of this inoculation, some degree of febrile indisposition is noticed, though the symptoms were seldom severe. But as these febrile paroxysms are said to have occurred, in some cases, as early as the third day after inoculation, and in others to have been protracted, without any evident cause, to the eleventh or twelfth day, or even later, a doubt arises whether we should consider a degree of fever as essential to the disease, or merely as an accidental occurrence.

Infants

Infants are ſo liable to febrile commotions of the ſyſtem, which ariſe from cauſes ſeldom very obvious, that in many of the inſtances above recorded, the exacerbations may have fallen in with a certain period of the Vaccine inoculation. The change intended to be produced in the ſyſtem by this diſeaſe, certainly implies that ſome conſtitutional affection ſhould be produced; but whether this may take place, in the generality of caſes, obſcurely, and without any evident diſturbance of the functions, does not appear perfectly clear. This circumſtance is of ſome importance, as we know the Cow-pock is capable of exciting a local diſeaſe in thoſe who have undergone the Small-pox, and of producing a fluid which is able to propagate the genuine Vaccine diſeaſe in ſuch as have neither been variolated nor vaccinated. It therefore becomes deſirable to diſtinguiſh ſuch caſes where only a local, or where a conſtitutional diſeaſe has been produced. In all the exanthemata, the eruption, which may be regarded as the criterion of a conſtitutional change having taken place, pretty regularly occurs on or before the fourth day; and in the inoculated Small-pox, where the analogy is more evident, the puſtules, if there be an eruption, uſually appear on the tenth. In theſe inſtances, fever may be regarded as conſtituting the eſſence of the diſeaſe. When failures have occurred in the inoculated Small-pox, they have generally ariſen from conſidering the ſeverity of the local affection as a ſufficient protection againſt the recurrence of the diſeaſe, without attending to the febrile ſtate of the ſyſtem.

It has been ſuppoſed that a certain number of perſons remain through life unſuſceptible of Small-pox infection; and the proportion has been computed to be about one in ſixty: It appears to me, however, more probable, that in many of theſe ſuppoſed inſtances of want of ſuſceptibility, the patients have undergone the diſeaſe during infancy, and have ſuffered the eſſential change operated by variolous fever, though no eruption appeared. A child about eight months old, which had always been very healthy, was inoculated in both arms with recent variolous matter, with which ſix other children were inoculated and experienced a favorable diſeaſe. Upon this child, however, the infectious matter produced no viſible effect, and the inciſed parts healed immediately. Three months afterwards the child was inoculated a ſecond time with fluid Variolous matter, introduced in as large a quantity as poſſible, but with as little effect as in the firſt attempt. Not being perfectly ſatisfied with the ſafety of the child, the inoculation was repeated at the end of ſix months from the laſt attempt, with matter of Cow-pock. Two punctures were made in each arm, and fluid Vaccine virus introduced into each. On the ſecond day the punctures punctures appeared red; the erythema increafed flightly until the fifth or fixth day, when it appeared to have attained its acmé. A ftigma or fmall elevated point now appeared in the centre of each puncture, and the rednefs gradually vanifhed. This might be given as an inftance of unfufceptibility of the difeafe; but to me it appears more probable, that the child had gone through the natural Small-pox at a more early period. The variolous fever had probably been fo flight as not to be noticed, or perhaps had been referred to cold, bowel complaints, &c. In fuch inftances, the eruption of a few folitary puftules in a very young infant, which do not maturate, are very apt to be referred by nurfes, like every other eruption, to the red gum. I have known itch puftules miftaken for the red gum. In inoculated Small-pox, fever is frequently the only vifible effect produced in the fyftem; and in many inftances of fcarlatina, no eruption appears. A fimilar inftance of meafles occurred to my obfervation, where it feems probable that the difeafe had run its courfe without any eruption. An infant, during the month, became affected with a flight degree of fever, heavinefs, and watering of the eyes, accompanied with a peculiar kind of cough, which continued fevere for a few days, and then abated. About three or four months afterwards the meafles were epidemic in the town, and three children in this family were feverely affected with the difeafe; in one, the fymptoms ran fo high as to threaten danger, but the infant, though expofed to the contagion, did not take the complaint. In the inftances of inoculated Cow-pock which fell under my care, upwards of fifty in number, including fubjects from four months to feventeen years of age, not a fingle inftance of indifpofition occurred which could be attributed to the inoculation. Thofe patients who were old enough to defcribe their fenfations, complained merely of forenefs and ftiffnefs in the axillæ.

The few folitary inftances which the opponents of the new practice have adduced as proofs of the inefficiency of the Vaccine difeafe to refift the contagion of Small-pox, have been, perhaps with propriety, referred to a *fpurious Cow-pock* introduced by miftake inftead of the genuine difeafe. If the fpurious Cow-pock, as it is termed, be a fpecific difeafe capable of producing a certain train of appearances in regular order, and in an indefinite number of fubfequent inoculations, there may be fome difficulty in detecting it when introduced into the human fyftem; but, on the contrary, if the effect produced by *the fpurious matter* be no more than a degree of erythema, fuch as would follow the infertion of any other acrid matter, and fuch as fometimes arifes in an unfuccefsful Variolous Inoculation, it is is improper to ufe the term *spurious Cow-pock*, as there is evidently no affinity between it and genuine Cow-pock. It has been faid, that the matter of genuine Cow-pock becomes decompofed by long keeping, and generates a fpurious difeafe. To this it may be objected, that Vaccine Virus has been fent to very diftant parts of the globe, to America and to the Eaft Indies, where it has communicated the genuine difeafe; and a furgeon of eminence affured me, that he had fucceeded perfectly well with Cow-pock matter which had been kept three months on the point of a common lancet. A doubt has been ftarted by fome who favour the new practice, left in procefs of time the Vaccine matter lofe its fpecific virus by being fucceffively propagated in the human fubject. This opinion, which experience alone can determine, is certainly deferving of attention. If the blue tinge which furrounds the puftule on the teat of the cow be the characteriftic fign of the genuine difeafe in that animal, and if it be obferved that this blue tinge is of a deeper hue in the human fubject, in proportion as it proceeds more immediately from the cow, it will give fome degree of probability to the above opinion, and alfo fhew the propriety of having occafional recourfe to the parent ftock. In all the cafes which fell under my care, the blue tinge furrounding the puftule was never fo intenfe as it is reprefented in fome of the plates of the Cow-pock, but appeared to be rather of a dirty white; this circumftance excited a degree of apprehenfion left the matter had not been genuine. The puftule affumed, in every inftance, the fame flat appearance, and was not much unlike a confluent Small-pock about the eighth or ninth day of the eruption, depreffed in the centre, elevated at the edges, which had a degree of tranfparency, and contained a pellucid fluid. The Cow-pock puftule having been called by fome writers a veficle, occafioned me to fuppofe that its contents would be evacuated inftantly by puncture, in confequence of which miftake the matter was loft on the firft trial. But, on a fecond attempt, it was found that by waiting a few feconds after the puncture was made, a drop like a pearl appeared to diftil from the orifice, and the fame takes place if feveral punctures be made. After the 12th or 13th day, the puftule began to change from the centre into a brown fcab, fomewhat larger than a fplit pea, or more nearly refembling a fplit horfe bean in form and colour. Thefe crufts, or fcabs, commonly continued two or three weeks. In no inftance did the inoculated part ulcerate, neither did the inflammation run to fuch a height as to require any attention, except in one child, where a faturnine lotion quickly ftopped its progrefs.

Some variety occurred in the extent of erythema round the inoculated inoculated part; in a few inftances there was only a faint blufh fcarcely exceeding the circumference of the puftule, while in others, inoculated with matter from the fame fource, the areola was of a fiery red, and equalled in fize a fhilling or half crown. There was alfo fome difference in the progrefs of the erythema; in fome the inoculated part was more advanced on the fixth day than in others on the ninth, in which laft inftances the puftules appeared very pale, and had only a trifling areola. In inoculating for the Small pox, I have been accuftomed to introduce as much matter as could conveniently be inferted on the point of a fpear-fhaped lancet, for which purpofe an incifion was made on each arm with a frefh lancet. This is contrary to the opinion of fome eminent practitioners, who advife a very fmall portion of matter, diluted with water, to be inferted; but having had reafon to be fatisfied with the former method, I have followed it in inoculating for the Cow-pox, and I have always ufed two armed lancets for the fame patient, making one or two punctures, an inch apart, in each arm. The mode of introducing the poifon was by inferting the point of a fine narrow lancet horizontally under the cuticle, until a portion of it was vifible through the cuticle; this was repeated a few times, until the whole of the virus appeared to be rubbed off. Notwithftanding the very rapid fuccefs which has attended the introduction of the Cow-pock, and notwithftanding it has triumphed over every oppofition, fome caution ought to be ufed in the choice of fubjects for inoculation, left an indifcriminate ufe of it in very young infants bring the practice into occafional difrepute, not from any danger attending the new difeafe itfelf, but from complaints incidental to infants, and which might be unjuftly attributed to the Vaccine difeafe. The inoculated Small-pox has no doubt fuffered from fuch caufes, as the following inftance may fhew. A woman applied to have a fine healthy child, about fix months old, inoculated from a neighbour's child in the natural Small-pox. The mother of the child, however, in the natural Small-pox, from a prejudice rooted among the vulgar, would not allow any matter to be taken, left her child fhould be hurt by the lofs of it. The other child, of courfe, was not inoculated; but on the third day after it ought to have been inoculated, the child was feized with convulfions, which continued four or five days, and carried it off. As there was no apparent caufe for the convulfions, they would have been charged upon the inoculation had it been performed.

In endeavouring to fpread the Cow-pock inoculation, medical men ought to be cautious how they depreciate the Variolous noculation, by afferting that it frequently excites fcrophula.

phula. The humoral pathology ſtill maintains its ground among the vulgar, who do not comprehend how one diſeaſe may bring another into action, unleſs it be by introducing the ſeeds of it into the ſyſtem.

A gentleman who had experienced the advantages of the Variolous inoculation in ſeveral of his children, obſerved, on reading ſome accounts recommending the Cow-pock, that there was an appearance of duplicity among medical men, as, until lately, theſe diſadvantages of inoculation were not *publicly* noticed.

*South Shields, June* 7, 1801.

